# Developing the Rationale for Including Virtual Reality in Cognitive Rehabilitation and Exercise Training Approaches for Managing Cognitive Dysfunction in MS

**DOI:** 10.3390/neurosci3020015

**Published:** 2022-04-03

**Authors:** Carly L. A. Wender, John DeLuca, Brian M. Sandroff

**Affiliations:** 1 Kessler Foundation, West Orange, NJ 07052, USA; cwender@kesslerfoundation.org (C.L.A.W.); jdeluca@kesslerfoundation.org (J.D.); 2 Department of Physical Medicine and Rehabilitation, Rutgers New Jersey Medical School, Rutgers University, Newark, NJ 07103, USA

**Keywords:** multiple sclerosis, cognition, cognitive rehabilitation, exercise training, virtual reality

## Abstract

Cognitive impairment is a common and detrimental consequence of multiple sclerosis (MS) and current rehabilitation methods are insufficient. Cognitive rehabilitation (CR) and exercise training (ET) are the most promising behavioral approaches to mitigate cognitive deficits, but effects are small and do not effectively translate to improvements in everyday function. This article presents a conceptual framework supporting the use of virtual reality (VR) as an ideal, common adjuvant traditional CR and ET in MS. VR could strengthen the effects of CR and ET by increasing sensory input and promoting multisensory integration and processing during rehabilitation. For ET specifically, VR can also help incorporate components of CR into exercise sessions. In addition, VR can enhance the transfer of cognitive improvements to everyday functioning by providing a more ecologically valid training environment. There is a clear interest in adding VR to traditional rehabilitation techniques for neurological populations, a stronger body of evidence of this unique approach is needed in MS. Finally, to better understand how to best utilize VR in rehabilitation for cognitive deficits in MS, more systematic research is needed to better understand the mechanism(s) of action of VR with CR and ET.

## 1. Introduction

Multiple sclerosis (MS) is an immune-mediated and neurodegenerative disease that affects upwards of 2.5 million adults worldwide [1]. The disease process results in multifocal central nervous system damage [2] and manifests as profound disability across physical and cognitive domains. Cognitive deficits, in particular, present in approximately 43–70% of persons with MS [3]. The most common cognitive deficits involve slowed information processing speed, impaired learning and memory, and executive dysfunction [4]. MS-related cognitive dysfunction results in burdensome, downstream consequences that permeate into everyday life and reduce quality of life [5]. Indeed, cognitively-impaired persons with MS are less likely to be employed, more vulnerable to psychiatric illness, and have difficulty completing everyday activities, such as shopping independently, completing household chores, driving, or navigating public transportation [3]. There is minimal, high-level evidence for the efficacy of disease-modifying therapies or symptomatic agents on cognition in this population, and there are no US Food and Drug Administration (FDA)-approved pharmacological treatments for cognitive impairment in MS [6]. Collectively, the high prevalence, debilitating consequences, and lack of effective pharmacotherapeutic treatments for cognitive impairment highlight the importance of considering other approaches for managing this consequence of the disease. Thus, rehabilitation has been proposed as a behavioral approach for mitigating MS-related cognitive impairment [7]. To date, the most promising approaches are cognitive rehabilitation (CR) and exercise training (ET).

## 2. CR and ET Approaches for Managing Cognitive Impairment in MS

### 2.1. CR

Beyond mitigating cognitive impairment, the ultimate objective of CR is to improve everyday function in individuals with cognitive deficits [8,9]. CR typically involves a team approach, wherein the individual with cognitive deficits, friends/family, and health care providers collectively identifies personally relevant goals of rehabilitation. Through individualized or group training with a provider, those goals are addressed by optimizing performance in preserved cognitive domains and integrating compensatory strategies to overcome persistent deficits. Cognitive training is a cornerstone of CR and involves guided practice on a set of standardized tasks that are designed to target specific cognitive domains (e.g., learning and memory) [8,10]. There is a growing convergence of evidence using neuroimaging paradigms in MS that CR may improve cognition via changes in brain function, such as activation and functional/effective connectivity in response to a task or at rest [7,11,12]. Additionally, emerging evidence supports small, but significant, downstream improvements on quality of life [13] and mental health symptoms (i.e., anxiety and depression) [14,15], which may help in managing cognitive impairment [16,17].

There is Class I evidence to support two CR techniques for persons with MS: the Kessler Foundation modified-Story Memory Technique (KF-mSMT) [18,19,20] as a practice standard for learning and memory deficits, and attention processing training (APT) [21] as a practice guideline for attention deficits [22]. This is based on randomized controlled trial (RCT) evidence indicating that the mSMT induces moderate improvements in learning and memory relative to a placebo control, as well as evidence supporting moderate-sized APT-related improvements in domains of attention relative to a placebo control [18,19,20,21]. Despite the aforementioned effects on specific domains of cognition, neither approach is associated with robust and sustained improvements in everyday functioning (although we do note some sporadic, immediate improvements on such outcomes) [18,19,20,21]. This is particularly noteworthy considering that transfer effects to everyday function is central to the purpose of CR [8]. Therefore, there is an opportunity for the provision of an adjuvant to successful CR approaches (i.e., mSMT and APT) to strengthen their overall effects on cognition and to better facilitate the transfer of intervention effects on proximal cognitive outcomes to distal everyday function outcomes.

### 2.2. ET

ET is defined as chronic, structured physical activity behavior with the purpose of improving or maintaining aspects of physical fitness. ET represents an alternative approach for managing cognitive deficits and downstream consequences of MS based, in part, on abundant evidence supporting ET-related benefits on cognition and the brain in older adults from the general population [23,24]. Unlike CR, ET does not involve activities to target specific cognitive domains or integrate behavioral strategies to adaptively compensate for cognitive deficits and their effects on everyday life. Rather, the physiological regulation of exercise behavior involves numerous interconnected neuropsychological processes that are also critical for cognitive performance [25]. Therefore, ET represents a potent and powerful behavior that has been hypothesized to improve cognition indirectly [25].

Compared with research on CR for managing cognitive dysfunction in MS, the evidence base regarding ET is substantially smaller and is in its relative infancy [25]. Overall, there is not yet Class I evidence to support any one ET approach for improving cognition in MS, largely due to methodological concerns of individual studies. For example, the vast majority of RCTs have not recruited persons with MS who are pre-screened for cognitive impairment [26,27] and have involved generalized ET stimuli of short duration [28]; such ET interventions are not based on neurophysiological hypotheses for inducing specific cognitive improvements based on specific neural mechanisms-of-action, and thus involve largely exploratory cognitive [29] and neuroimaging endpoints [26,30]. Despite the overall issues, in recent years, the methodological quality of studies has generally improved [31,32]. Several small studies support ET-related benefits on domains of cognition typically affected by MS [7,33,34] as well as on brain structure, function, and connectivity in MS [35] and quality of life [36]. Nevertheless, while not yet considered a standard-of-care, given the lack of Class I evidence, the recent proliferation of lower-level evidence positions ET as a promising approach for managing MS-related cognitive impairment [25]. There further is a dearth of evidence on ET-related improvements in downstream everyday functioning as a result of cognitive improvements in persons with MS [25,37]. As is the case for CR, traditional ET could greatly benefit from an adjuvant that strengthens overall effects on cognitive outcomes that better transfer to downstream improvements in everyday functioning.

## 3. Enhancing CR and ET Approaches for Managing Cognitive Impairment in MS

Although considered the two strongest approaches for managing MS-related cognitive impairment, neither CR nor ET completely addresses this devastating manifestation of the disease. As described above, there are two areas associated with each approach that can be addressed via inclusion of a common adjuvant: (1) overall small-to-moderate effects on cognitive outcomes; and (2) lack of robust transfer effects on everyday function outcomes [25].

The notion of supplementing rehabilitation approaches with at least one adjuvant is not new, as researchers and clinicians acknowledge that highly individualized, multimodal rehabilitation techniques are likely required to effectively overcome challenges such as MS-related cognitive dysfunction [38,39]. However, there are no conceptual frameworks to implement such a multimodal rehabilitation technique for maximizing cognitive outcomes in persons with MS. Such a framework could be particularly useful considering that implementing a common adjuvant for CR and ET paradigms for improving cognition in MS might strengthen effects of cognitive and everyday function outcomes in response to both approaches.

In this paper, we present the conceptual argument that virtual reality (VR) represents an ideal, common, adjuvant to enhance the effects of traditional CR and ET on cognitive outcomes in clinical trials involving persons with MS. We initially present an argument for VR allowing for enhanced effects of both approaches on cognition in persons with MS. We then present an argument for VR being conducive for facilitating the transfer of such cognitive improvements into downstream improvements in everyday function in persons with MS. Finally, we provide a roadmap for future research examining the implementation of VR with CR/ET in research for managing MS-related cognitive dysfunction.

## 4. VR as an Adjuvant for CR and ET Approaches for Cognitive Impairment in MS

VR involves the use of advanced technologies to create a simulated (i.e., virtual) environment that users perceive as analogous to the real world [40]. VR can be categorized in terms of immersion and interaction. Immersive VR refers to approaches wherein a user wears a headset such that their point of view is entirely contained within the virtual environment (e.g., Oculus Quest) [41]. Conversely, non-immersive VR refers to approaches wherein a virtual environment is placed within or around the real world and a user can experience the virtual and real-world environments simultaneously (e.g., X-Box Kinect). Interaction is a user’s degree of control [41], and a VR program can range from entirely interactive (e.g., full body tracking) [42] to completely non-interactive or passive (e.g., automatic movement through a virtual environment) [43]. Together, immersion and interaction relate to a greater sense of presence (i.e., the feeling of really being there) [44] and engagement within the virtual environment [45,46]; those elements are conducive for inclusion with CR and ET approaches for managing cognitive impairment in MS (Table 1).

While VR first surfaced in the 1960s [55], there has been a recent renaissance in its usage as technology has improved, costs have decreased, and availability has risen in kind [47]. VR has become a popular tool in pain management [56,57], exposure-based treatment for anxiety [58,59], phobias [60,61], and post-traumatic stress disorder [62,63], treatments for substance abuse disorders [64,65], and physical rehabilitation [66,67] in a wide range of populations. Of note, VR was applied in these cases under the presumption that, compared to traditional treatment methods, VR-based methods would be more stimulating and engaging [68] and would more closely mirror real-world scenarios [47], which would in turn relate to greater treatment effects [47,69]. For similar reasons, there is a growing interest in utilizing VR to improve psychological well-being and cognition (i.e., memory) in older adults with mild cognitive impairment, dementia, and Alzheimer’s disease. The overall evidence is difficult to summarize due to small sample sizes, uncontrolled designs, and heterogeneous results, though there is clear support for the feasibility [70] and enjoyment [71] of VR in these populations wherein future experiments are warranted [72,73]. By extension, we hypothesize that the inclusion of VR within CR/ET paradigms can enhance cognitive improvements that further result in improved everyday functioning among persons with MS.

### 4.1. VR for Enhancing Cognitive Improvements

VR is a well-equipped adjuvant to strengthen the effects of CR/ET by substantially increasing sensory input and promoting multisensory integration and processing during rehabilitation [74]. Multisensory processing during learning or cognitive training is linked to better memory for unisensory objects, better performance on simple cognitive tasks, and improved performance on more complex, higher-level tasks [74,75]. Therefore, incorporating multisensory feedback and integration into CR/ET via VR should induce larger cognitive improvements. For example, traditional CR/ET interventions in MS are highly controlled and typically take place in a mundane laboratory with few distractions. By comparison, using VR, someone could be placed into an expansive virtual environment depicting a visually stimulating cityscape surrounded by colors and shapes, along with auditory stimuli from the traffic on the streets or the people on the sidewalks. Such an environment could include audio-visual stimuli from electronic billboards down the street or haptic stimuli of steam coming up from the underground subways. As the complexity of a virtual environment increases, cognitive stimuli can become more challenging, conceptually leading to greater adaptations and stronger intervention effects over time with repeated exposure.

The above argument is supported by studies that apply VR to traditional rehabilitation for paralysis due to stroke. Evidence indicates that VR-based mirror therapy is superior to traditional mirror therapy on motor outcomes of the affected limb due to an increased volume of visual stimuli [76] and the inclusion of multisensory feedback (e.g., auditory, audio-visual, proprioceptive) [77,78]. The larger improvements in motor outcomes further are explained by greater neural activation in the affected motor areas from VR-based mirror therapy [77,78,79]. In this case, VR-based therapy led to greater neural activation in the region-of-interest than traditional rehabilitation. By extension, VR-based CR/ET could lead to greater neural activation in brain regions-of-interest than traditional CR/ET, potentially resulting in substantial cognitive improvements.

#### 4.1.1. VR for Strengthening Effects of CR

One previous study of VR-based CR in persons with MS supports the hypothesis that VR administration increases the volume (i.e., perceptual load) of visual stimuli [50] which leads to strengthened cognitive outcomes [54,80]. Participants (*n* = 30) were randomized to either traditional, face-to-face, individual sessions of CR (i.e., control group) or CR conducted through the VR rehabilitation system (VRRS), an internationally patented Class I certified medical device. The VRRS allowed individuals to investigate and manipulate objects using simple hand movements. Greater visual sensation was achieved in VR with 3D objects and their interactions within a detailed, naturalistic environment, compared to the standard paper-and-pencil cognitive training employed in the traditional CR control group. There were significantly greater benefits of the VR-based CR program on global cognitive function, learning ability and verbal short-term memory, lexical ability, and quality of life [81].

There is emerging evidence to support the relationship between multisensory processing in VR and cognitive improvement in MS. A popular implementation of VR for neurologic populations is in driving simulators [82,83,84,85], but most studies evaluate driving-specific outcomes rather than performance on traditional neuropsychological assessments. However, one study demonstrated that, compared to a no treatment control group, a VR-based driving simulation improved processing speed (PASAT) in individuals with MS [86]. Such improvements support the conceptual hypothesis that perhaps increased sensory stimuli and multisensory processing during a VR-based driving simulator required more efficient neural communication in regions associated with processing speed than traditional CR, leading to larger cognitive improvements over time. In another study (*n* = 60), traditional CR was administered using a non-immersive VR program called “BTS-Nirvana”. Similar to the VRRS, BTS-Nirvana allowed individuals to manipulate virtual objects in a detailed, real-world environment. Unlike the VRRS, BTS-Nirvana included audio-visual stimuli and multisensory feedback. Compared to those randomized to a conventional CR condition (i.e., control), individuals in the VR condition showed greater improvements in visual perception, visuospatial abilities, short term visual memory, working memory, executive functions, speed of information processing, and sustained attention [87].

Collectively, VR can enhance the effects of CR by increasing the volume of visual stimuli provided during training and by integrating multisensory stimuli to promote more complex multisensory processing during training in persons with MS [88]. Limited evidence comparing VR-based CR to traditional CR supports continued investigation of conducting cognitive training within a 3D, rich, detailed virtual environment to strengthen cognitive effects of traditional CR in this population.

#### 4.1.2. VR for Strengthening Effects of ET

The provision of a myriad of sensory stimuli in VR is conducive for enhancing the effects of traditional ET on cognition, as is the case for CR. Multisensory processing is the backbone of the PRIMERS model, which hypothesizes that cognitive benefits of ET in persons with MS stem from the integration and processing of visual, vestibular, locomotor, proprioceptive, cardiopulmonary, pulmonary, thermoregulatory, and endocrine stimuli [89]. However, no study to date has investigated the addition of VR to ET for enhancing cognitive improvements in MS within the context of the PRIMERS model.

From a conceptual standpoint, VR allows for the incorporation of components of CR (i.e., training preserved cognitive domains and integrating compensatory strategies) into exercise sessions, which could provide a more direct approach of ET for improving specific cognitive domains (Table 1). This is particularly advantageous considering that ET is hypothesized to improve cognition indirectly [36]. Such a VR-based approach for cognitive training can be mapped to ET prescriptions, given that both ET and cognitive training are typically highly regimented and repetitive interventions. In addition, as VR is associated with greater cognitive engagement (i.e., presence) and coordination of sensorimotor systems by providing multisensory stimulation (i.e., visual, proprioception) [90,91], its inclusion in ET paradigms increases the likelihood of larger cognitive improvements than ET alone over time. That hypothesis further aligns with the notion that such multisensory stimulation can lead to greater rehabilitation-related activation in a wider range of brain regions [92], which likely translates to greater cognitive improvements over time [89]. Indeed, those hypotheses are supported by evidence in older adults with mild cognitive impairment whereby CR and ET with VR was associated with larger cognitive improvements than traditional CR and ET [93].

Collectively, consistent with the application of VR to CR, VR can enhance the effects of ET by promoting greater multisensory processing. Further, from a conceptual standpoint, the addition of VR can help incorporate aspects of CR into traditional ET which could induce larger, domain-specific cognitive improvements than traditional ET. However, evidence-based support for those hypotheses in persons with MS is necessary, as no studies have directly tested the addition of VR to traditional ET for such purposes.

### 4.2. VR for Enhancing Translation to Improvements in Everyday Function

In addition to strengthening overall effects on cognitive functioning, the inclusion of VR is advantageous for enhancing the transfer of cognitive improvements to improvements in everyday functioning. VR uniquely allows for the ability to conduct tightly controlled clinical trials involving rehabilitation in settings that more closely mirror the real-world (i.e., improved ecological validity). Such enhancements in ecological validity conceptually allow for better translation of cognitive improvements to downstream improvements in everyday functioning [94,95]. The real world is complex, uncontrollable, messy, and is not conducive for early-stage RCT research that must balance ecological validity with internal validity. Comparatively, VR allows for precision over every event that happens in a virtual environment and allows time to be stopped and rewound, enabling for real-time feedback and enhanced practice [96,97]. The working assumption is that by training and improving cognitive skills in a simulated real-world scenario, the similarities between the learning environment and actual environment will facilitate more effective translation to improvements in cognition and everyday function using well-validated outcomes [98,99,100].

Relatedly, the true potential of VR is in the high level of control that experimenters have over every aspect of the environment while matching the complexity of the real world. Indeed, the continuum of virtual environments spans from simplistic and unisensory focused, that can target one specific cognitive function, to a complex and multisensory environment to broadly and concurrently address multiple cognitive domains. Every sense and stimulus can be controlled or programmed by an experimenter, to balance challenges with frustrations, while providing sufficient experiences of success and accomplishment using dynamic adjustment to encourage continuation [101,102]. Importantly, this applies for both CR and ET approaches for improving the translation of cognitive improvements to improvements in everyday functioning in persons with MS.

#### 4.2.1. VR for Everyday Functioning in CR

Although two published studies included VR with CR for inducing cognitive improvements in persons with MS that incorporated realistic environments and movements in the VR stimulus for improving ecological validity [81,86], neither measured long term outcomes related to everyday functioning. By comparison, in other neurological populations, there is evidence supporting multiple CR approaches that incorporate VR (and high ecological validity) for inducing significant benefits on perceived cognitive deficits pertaining to everyday life [103,104].

#### 4.2.2. VR for Strengthening Effects of ET

Ecologically valid ET involves more natural forms of exercise, such as outdoor cycling, basketball, or other sports, compared to strictly regimented indoor aerobic or resistance ET interventions. In healthy adolescents, healthy adults, and persons with schizophrenia, such ecologically valid ET approaches have demonstrated significant immediate and longer-lasting effects on a broad range of cognitive domains including global functioning, executive function, spatial abilities, and working memory [105,106,107,108]. We note that although such ecologically valid approaches are advantageous for inducing improvements in everyday functioning, these are difficult to control in a supervised, laboratory-based setting, which is critical for maintaining internal validity for initial evaluations of efficacy of ET. This underscores the importance of including VR for such a purpose. To date, there have been no ET studies that have included VR for creating an ET stimulus with high ecological validity for inducing cognitive and everyday functional improvements in persons with MS.

## 5. Roadmap for Testing the Conceptual Framework in Clinical Trials

VR represents an ideal, common, adjuvant to enhance the effects of traditional CR and ET on cognitive outcomes in persons with MS. Conceptually, VR can strengthen benefits of CR by increasing the volume of sensory stimuli and promoting multisensory integration and processing in persons with MS. VR can also strengthen the benefits of ET by promoting multisensory integration and processing as well as by incorporating aspects of traditional CR for a more targeted approach to strengthen ET-related effects on cognitive outcomes in those with MS. VR can also potentially better promote the transfer of such cognitive effects of CR and ET to everyday functioning in persons with MS by increasing real-world relevance and ecological validity. Given such a conceptual framework for including VR as an adjuvant to high-quality CR and ET approaches for improving cognition and everyday functioning in MS, we now provide a roadmap for how this conceptual framework can be tested in clinical trials in MS.

Although there is a clear interest in using VR to enhance traditional approaches for managing cognitive impairment in neurologic populations, a stronger body of empirical research is needed to better understand the mechanism(s) of action and most important characteristics for optimizing future interventions and clinical trials in persons with MS, given that the advantages of VR for enhancing cognition are largely conceptual. Future research efforts are needed that focus on the implementation of VR with CR/ET can systematically manipulate characteristics of VR (Table 1) for strategically inducing specific cognitive improvements and their downstream effects on everyday functioning. Indeed, identifying and matching key ingredients of the CR/ET and VR prescriptions based on hypothesized mechanisms of action and the targeted outcomes can lead to a powerful approach for rehabilitation that is clinically relevant.

Immersive VR is safe and feasible to use in persons with MS [109] and can be employed more to strengthen the effects of CR by increasing sensory input. Studies to date have utilized non-immersive VR in MS. However, there is evidence suggesting that immersive VR is associated with greater engagement and presence, thus increasing the likelihood of observing cognitive improvements [110,111]. Using the aforementioned conceptual framework, the application of VR is highly conducive for strengthening cognitive improvements associated with APT in persons with MS. For example, in traditional APT, one task involves an individual role playing as a train conductor who completes related tasks using a computer program controlled by a mouse and keyboard [112]. This traditional CR approach could be compared to one in which APT is delivered through immersive VR, such that the experimenter can “place” a participant into a virtual train and provide the participant with controllers with which they can grab levers and push buttons in the control room. The experimenter further can place a virtual co-conductor next to the participant who provides instructions on the participant’s task to transport a group of people across the country safely. Conceptually, such a VR environment provides a far larger degree of multisensory stimuli than traditional APT, which might lead to stronger improvements in attention. The level of multisensory input from VR in such an approach further can be manipulated. Early-stage experiments might only provide increased visual and auditory stimuli, similar to sensory perception in the traditional APT, and if successful, later experimental research could incorporate additional sensory input or distractors in the VR to present a greater challenge to induce improvements in domains of attention. Including appropriately difficult stimuli during cognitive training and progressively increasing the challenge as the individual progresses is a key ingredient to the success of CR [8].

To test the combination of ET and VR for inducing cognitive improvements in persons with MS, one could add a very simple virtual environment to a treadmill walking intervention, such as the individual’s favorite park or a country they want to visit. This could provide additional multisensory input which could result in greater cognitive benefits over time, as per the PRIMERS hypothesis [89]. Consistent with the aforementioned example for CR, the next step could involve manipulating immersion by presenting the virtual environment either on a 2D screen (i.e., non-immersive) or through a VR headset (i.e., immersive). Alternatively, one could compare interactive VR, where a person’s walking speed dictates the speed at which they move through the virtual environment, to a non-interactive, or passive, VR, where the speed of movement in the virtual environment is locked and disconnected from actual walking speed for differentially inducing cognitive improvements. However, such an endeavor would need to carefully consider ambulatory status as a potential factor [113]. To use VR to add components of CR to ET interventions in persons with MS, one could add in training of cognitive skills from mSMT to aerobic cycling ET via VR by adding a story narrative to the VR and instructing individuals to pay special attention to imagery and context while cycling at a certain exercise intensity that is likely to elicit aerobic fitness benefits over time.

It is not easy to create an ecologically valid VR program, which might explain why very few studies have combined ecologically valid VR programs with CR or ET. This may further explain the complete lack of research on VR as an adjuvant for CR or ET for inducing everyday functional improvements in persons with MS. Of note, VR programs have been created for cognitive assessment (including in persons with MS [114,115]), and might be conducive for application in rehabilitation settings. Such approaches are particularly exciting for application with established CR and ET approaches in eventual clinical trials on cognition and everyday functioning in this population. By comparison, there are commercially available VR programs that combine with ET (e.g., Octonic for walking or VZFIT for cycling) to create a more ecologically valid experience. We note that several pilot studies have successfully combined treadmill walking with non-immersive VR in persons with MS [116,117], supporting safety and feasibility for such an intervention, though such pilot trials have not included cognitive or everyday functioning measures. Nevertheless, the consideration of such approaches for inducing improvements in cognition and everyday function is warranted.

The advantages of combining VR with CR and ET for inducing larger cognitive improvements than traditional CR/ET that translate to more robust improvements in everyday functioning in persons with MS are largely conceptual. Given the overall dearth of evidence in this area, future research efforts might consider initially adopting non-RCT designs prior to immediately investing considerable time and resources into a large RCT. Although such early-stage, non-RCT designs are associated with Class III or below evidence, such research might provide the foundation for a particularly strong trial that can eventually provide Class I evidence. For instance, researchers might consider adopting within-subjects, repeated-measures designs for initially comparing different VR/CR/ET parameters for optimizing cognitive and everyday functional improvements that can be included in a RCT. This could be followed by single-group, pre/post designs as proof-of-concept research for improving cognition/everyday function over time with a combined VR plus CR/ET intervention. Such quasi-experimental research could then inform the design of an early-stage RCT that involves a passive control condition for demonstrating feasibility, followed by efficacy testing that could compare the effects of combined VR plus CR/ET with each approach alone in larger MS samples. We further note that such approaches have been used successfully in both CR and ET research among persons with MS [25]. Lastly, regardless of the stage of research, such trials should carefully consider demographic and clinical characteristics of MS samples that might predict particularly beneficial responses to a combinatory approach, given evidence of response heterogeneity in CR and ET research on cognitive outcomes in this population [7].

## 6. Summary/Conclusions

VR represents a strong candidate as a stimulating, engaging, complex, and ecologically valid addition to conventional CR and ET approaches for managing MS-related cognitive impairment and its consequences. Such a combinatory approach is advantageous for facilitating enhanced cognitive improvements relative to traditional CR/ET approaches alone as well as for increasing the likelihood that such interventions will translate to long-term, downstream improvements in everyday function. More research is clearly warranted to investigate the efficacy of combining VR with CR and/or ET for managing cognitive impairment and its downstream consequences on everyday functioning in persons with MS [10]. Such research endeavors can test a conceptual framework using several different approaches for advancing this field to ultimately improve the lives of those with the disease.

## Figures and Tables

**Table 1:** VR components that are conducive for inclusion in CR and ET interventions on cognition in MS.

VR Component	Definition	Application for CR	Application for ET
Immersion	A user experience where the real world is shut out and a user is surrounded by a virtual environment [47]. The virtual environment changes in a natural way with head and body motion, similar to that in the real world [48].	Greater engagement/presence and sensory stimulation by implementing CR techniques and characteristics of cognitive training into a virtual environment, increasing the likelihood of cognitive improvements.	Greater engagement/presence and sensory stimulation by creating a multisensory experience during ET, increasing the likelihood of cognitive improvements.
Interaction	The ability of a user to make changes in and control aspects of the virtual environment [47]. Interaction can come from hand controllers, eye-tracking, natural locomotion, and full body tracking [47].	Increases engagement/presence by engrossing individuals in the environment and increasing their sense of control. Interaction provides more realistic practice in cognitive training.	Increases engagement/presence by engrossing individuals in the environment and increasing their sense of control. Interaction with the VR can be tightly matched with actual physical movement.
Presence	The feeling of “being there” in a virtual environment [44,49].	The key ingredient for engagement in a virtual environment. Presence is also linked to greater motivation to complete tasks in VR and fun doing it.	The key ingredient for engagement in a virtual environment. Presence is also linked to greater motivation to complete tasks in VR and fun doing it.
Naturalistic environment	A virtual environment that resembles the real world.	Can resemble the complexity of the real world and provide more ecologically valid training. Potential for CR to train more high-level cognitive domains important for everyday function.	Increases engagement and enjoyment of ET. Can provide multisensory input that more closely resembles the real world, increasing the likelihood of cognitive improvements that translate to improvements in everyday function.
Perceptual load	The number of objects present in a virtual environment that may or may not be targets [50]	Low perceptual load environments may be helpful in initial stages of training [51,52] but increasing perceptual load over time can create challenges and improved cognitive performance [53,54].	Perceptual load can increase multisensory processing required during ET and the cognitive challenge during ET.
Individualization	The ability to change aspects of the virtual environment to precisely match the individual’s need to desire.	Can resemble the complexity of the real world and provide more ecologically valid training. Potential for CR to train more high-level cognitive domains important for everyday function. Can motivate someone to work harder and can be much more enjoyable due to the personal connection to the VR. The challenge of the task can change specifically with regard to individual performance. Ideally suited for laboratory-based clinical trials. Specific and individualized feedback can be provided.	Increases engagement and enjoyment of ET. It may also help translate ET to higher levels of leisure time physical activity. Can motivate someone to work harder and can be much more enjoyable due to the personal connection to the VR. Individualization can help target specific cognitive domains to improve upon during ET. Ideally suited for laboratory-based clinical trials.
Time Manipulation	VR does not follow the rules of reality allowing examiners to pause, slow down, and rewind scenarios presented in the virtual environment.	Allows for more specific, real-time feedback which enhances training.	Allows for more specific, real-time feedback which enhances training.

## Data Availability

Not applicable.
